# Exploratory study of clinical effectiveness and safety of TJ-116 bukuryoingohangekobokuto for anxiety and postoperative water brash in esophageal cancer patients (TJ116E)

**DOI:** 10.1097/MD.0000000000020317

**Published:** 2020-05-29

**Authors:** Ryutaro Arita, Shin Takayama, Hiroshi Okamoto, Ken Koseki, Yusuke Taniyama, Soichiro Kaneko, Rie Ono, Satoko Suzuki, Takashi Kamei, Tadashi Ishii

**Affiliations:** a Department of Kampo Medicine; b Department of Education and Support for Regional Medicine, Tohoku University Hospital; c Department of Kampo and Integrative Medicine, Tohoku University Graduate School of Medicine; d Department of Surgery, Tohoku University Hospital; e Department of General Practitioner Development, Tohoku University School of Medicine, Sendai, Japan.

**Keywords:** anxiety, aspiration, bukuryoingohangekobokuto, esophageal cancer, Kampo medicine, randomized controlled trial, traditional Japanese medicine

## Abstract

**Background:**

Patients with esophageal cancer suffer from anxiety in the perioperative period surrounding esophagectomy; this may increase the risk of postoperative complications. In particular, postoperative aspiration pneumonia carries a high risk of hospital mortality. Bukuryoingohangekobokuto (BRIHK) is a traditional Japanese medicine formula used to treat anxiety, the feeling of a foreign body in the esophagus, and water brash. We hypothesize that BRIHK might be effective for both anxiety and water brash in perioperative patients with esophageal cancer. The aim of this study is to evaluate the efficacy and safety of BRIHK compared to a placebo for anxiety and water brash in perioperative esophageal cancer patients.

**Method/design:**

This will be a single-center, single blind, placebo-controlled randomized clinical trial. Twenty-four patients with esophageal cancer undergoing radical resection surgery will be registered to participate, then randomly and blindly assigned to the BRIHK treatment group or control group. Patients will be administered BRIHK or the placebo from 2 weeks before to 6 weeks after surgery. Primary outcome measures will be anxiety and depression (assessed using the Hospital Anxiety and Depression Scale), and water brash (assessed using the 10-item Eating Assessment Tool, Esophagus and Stomach Surgery Symptom Scale, and videofluoroscopy swallowing measurement). Incidences of aspiration pneumonia will be noted and abdominal gas volume, inflammatory markers, and nutrition status will be evaluated.

**Discussion:**

This investigative study will provide clinical evidence of BRIHK administration for anxiety and water brash, which might improve mental distress and reduce postoperative mortality.

**Trial registration:**

The protocol and progress are registered on the Japan Registry of Clinical Trials (jRCT s021190001) and University Hospital Medical Information Network (UMIN000031330). The protocol was approved by the Japanese Ministry of Health, Labour and Welfare certified clinical research review board, Tohoku University (CRB2180001).

## 1 Introduction

Patients diagnosed with fatal cancer have a greater risk of developing anxiety than the general population.^[1]^ In patients referred for surgery, the most common factors contributing to anxiety are concern about family and fear of complications, results of the operation, and postoperative pain.^[2]^ Preoperative anxiety is associated with increased anesthetic requirements,^[3]^ increased pain,^[4]^ nausea and vomiting,^[5]^ and prolonged hospitalization^[6]^ in the postoperative period. Previous articles have reported that 34% of patients with esophageal cancer have possible or probable anxiety prior to surgery,^[7]^ and 16% to 34% of postoperative patients still have anxiety during the follow-up year after diagnosis.^[8]^ Anxiety is a clinical issue in the perioperative period.

Esophagectomy is a standard surgical procedure for the treatment of esophageal cancer. Its morbidity and mortality rates after surgery have decreased in recent decades, but remain higher than those of other thoracic surgical procedures.^[9]^ In particular, postoperative pneumonia is one of the most common and fatal complications after surgery, occurring in 9% to 19% of patients even in recent reports,^[10–13]^ and is a major risk factor of hospital mortality. In a videofluoroscopic swallowing study, 32% of postoperative esophageal cancer patients showed evidence of subglottic aspiration, which is an important preliminary risk of aspiration pneumonia.^[14]^ As such, prevention of postoperative pneumonia is a clinical issue in esophageal cancer surgery.

Together with Western medicine, traditional Japanese (Kampo) medicine has been widely used in Japan for the treatment of both mental and physical disorders. Bukuryoingohangekobokuto (BRIHK) is used for patients with anxiety, depressed feelings, the feeling of a foreign body in the throat and esophagus, nausea, heartburn, nervous gastritis, water brash, and more.^[15]^ Hangekobokuto, which is included in BRIHK, has been reported to improve the impaired swallowing reflex^[16]^ and reduce the risk of pneumonia and pneumonia-related mortality in clinical trials of elderly patients.^[17]^ A recent trial showed hangekobokuto prevented aspiration pneumonia in patients undergoing cardiovascular surgery.^[18]^

We hypothesize that BRIHK administration in the perioperative period could alleviate anxiety and reduce water brash in patients who undergo esophagectomy. This protocol outlines a study aimed to investigate the efficacy and safety of BRIHK for anxiety and water brash, that is, aspiration during the postoperative period in esophageal cancer patients.

## 2 Methods/design

### 2.1 Study design and setting

**Figure 1:**
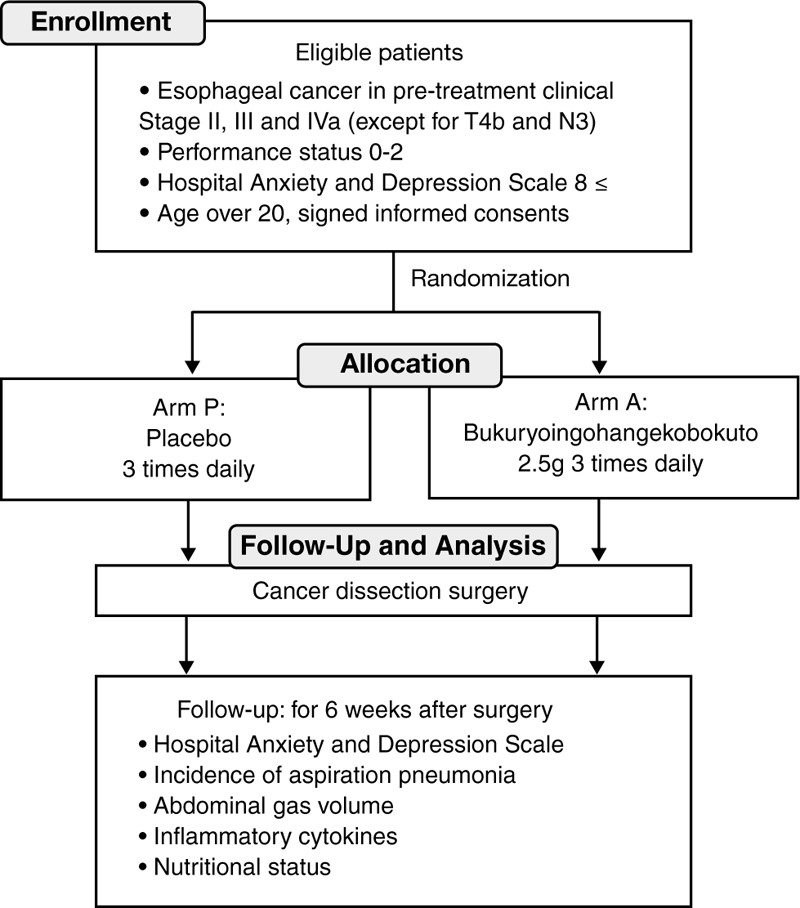
Study design: single-blind placebo-controlled randomized clinical trial.

The study will be a single-center, single blind, parallel randomized controlled trial to assess the efficacy and safety of BRIHK for anxiety and postoperative water brash in esophageal cancer patients. Participants will be blind to the treatment assignment throughout the trial. The study design is shown in Figure 1.

### 2.2 Eligibility

Those who meet all the following inclusion criteria and do not meet any listed exclusion criteria will be eligible to participate.

#### 2.2.1 Inclusion criteria

1. Scheduled to undergo elective radical resection surgery for esophageal cancer in the upper, middle, or lower parts
2. Pre-treatment clinical stage II, III, or IVa according to the 8^th^ edition of the Union for International Cancer Control TNM classification (except for T4b and N3)^[19]^
3. Eastern Cooperative Oncology Group performance status of 0, 1, or 2
4. Hospital Anxiety and Depression Scale (HADS) score ≥ 8^[20]^
5. Aged ≥ 20 years at the time of consent
6. Either sex
7. Provision of written informed consent

#### 2.2.2 Exclusion criteria

1. History of abdominal surgery (except for appendectomy or caesarean section) or ileus
2. Inflammatory bowel diseases
3. Undergoing emergent surgery for esophageal cancer
4. Concomitant use of prohibited medicine 4 weeks before inclusion
5. Past history of radiotherapy or chemotherapy for esophageal cancer
6. Allergy to Kampo formulas or intolerance to lactose (major excipients of Kampo formulas)
7. Severe liver, kidney, cardiovascular, blood, or metabolic diseases
8. Pregnant, within 28 days after childbirth, or breastfeeding
9. Dementia
10. Difficulty with study participation as judged by the researchers

Prohibited medicines in this study include Kampo formulas other than BRIHK and psychotropic agents. Temporary use of hypnotics will be allowed if needed.

### 2.3 Interventions

In study arm P (placebo group), 2.5 g of lactose powder (Pfizer Japan Inc, Tokyo, Japan) will be orally administered 3 times daily before meals from 14 days before surgery to 42 days after surgery. On the day of the operation, administration will be stopped. During the fasting period after surgery, lactose will be dissolved in warm water and administered through an intestinal tube via the transintestinal fistula. Lactate is set as a non-active ingredient.

In study arm A (BRIHK group), 2.5 g of BRIHK will be orally administered 3-times daily before meals on the same schedule and using the same methods as in arm P. BRIHK is a multicomponent formula, originally extracted from 9 crude drugs as follows: 6 g Japanese Pharmacopoeia (JP) Pinellia tuber, 5 g JP Poria sclerotium, 4 g Atractylodes lancea rhizome, 3 g JP Magnolia bark, 3 g JP Citrus unshiu peel, 3 g JP Ginseng, 2 g JP Perilla herb, 1.5 g JP Immature orange, and 1.0 g JP Ginger (JP: the 17th Japanese Pharmacopoeia^[21]^). It is currently prepared for prescription use in Japan as granules through the process of decoction, concentration, drying, and the addition of excipients (Tsumura BRIHK extract granules for ethical use, Tsumura & Co., Tokyo, Japan).^[15]^

We will place a label on both the packages of lactose and BRIHK for blinding. We will monitor the participants’ adherence by having them return unused medication.

### 2.4 Outcomes

The primary outcome measures include mental status and water brash before and after surgery. Mental status will be assessed using HADS, which contains both anxiety and depression subscale scores.^[15]^ Water brash will be assessed using the 10-item Eating Assessment Tool (EAT-10), Esophagus and Stomach Surgery Symptom Scale (ES4) and videofluoroscopy swallowing measurement. EAT-10 is a screening assessment scale for dysphagia.^[22]^ ES4 measures the postoperative symptoms of esophagogastric cancer.^[23]^

Secondary outcome measures include the incidence of aspiration pneumonia, number of postoperative hospitalization days, abdominal gas volume, plasma inflammatory markers, chest computed tomography findings, nutritional status, and laboratory examination findings. Aspiration pneumonia will be diagnosed based on the clinical practice guideline for pneumonia.^[24]^ Abdominal gas volume will be measured by analyzing an abdominal radiograph using Koide methods with Image J.^[25,26]^ White blood cell count, serum interleukin-6, tumor necrosis factor α, and granulocyte-colony stimulating factor will be measured as inflammatory markers. Serum albumin, prealbumin, total cholesterol, retinol-binding protein, transferrin, C-reactive protein, and lymphocytes will be measured as nutrition status markers. We will also evaluate body weight, body mass index, triceps skinfolds, and arm muscle circumference measured using bioelectrical impedance analysis (InBody 770, InBody, Soul, Korea).

Secondary safety measurements will include adverse events, side effects of BRIHK, and laboratory data abnormalities exceeding postoperative change. BRIHK has a possible side effect of hypersensitivity (e.g., rash or urticaria).^[15]^ We will routinely examine the laboratory data for postoperative adverse events such as those involving liver function, kidney function, electrolytes, creatine phosphokinase, glucose, and brain natriuretic peptide.

### 2.5 Participant timeline

**Figure 2:**
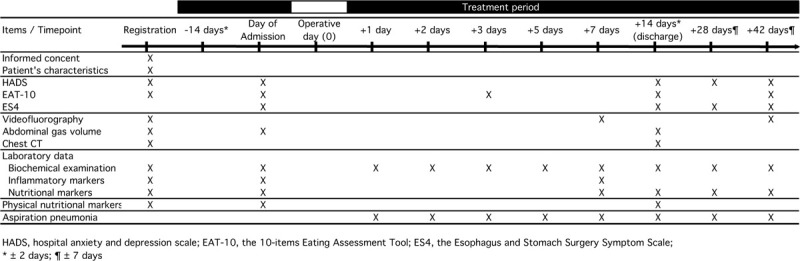
Time schedule.

The time schedule for this trial and data collection is summarized in Figure 2.

### 2.6 Sample size

There is no clinical evidence regarding the treatment effects of BRIHK for anxiety or water brash. Therefore, we will estimate the appropriate sample size based on feasibility instead of on statistics.

### 2.7 Recruitment

Twenty-four Participants will be recruited from outpatients in the esophagus-stomach surgery department at Tohoku University Hospital, Sendai, Japan.

### 2.8 Randomization, allocation, and blinding

Registration and allocation of the participants will be performed by the investigators and WDB Clinical Research Co., Ltd. The eligible patients will be blindly randomized to either arm P or arm A in a 1:1 ratio. The random sequence will be created using computer-generated permuted block randomization.

### 2.9 Data collection

All data will be collected and recorded on a paper-based case report form by the investigators. Patient characteristics, the questionnaires, and evaluation forms such as HADS, EAT-10, ES4, as well as the findings of videofluoroscopy swallowing measurement and CT, abdominal gas volume, laboratory data, and physical status will be collected. Adverse effects will also be recorded. After recording the data, the investigators will confirm the case report form, referring to its source documents.

### 2.10 Data management

We will assign all participants identification code numbers. Upon registering and recording the case report form, personal identifying information (e.g., name, address, phone number, medical record number) will be deleted from the data. The linkable anonymized number table will be created and stored by a personal information manager. All data will be kept in locked storage for at least 5 years after study completion. The data management center (WDB Clinical Research Co., Ltd.) will confirm the case report form to check for duplication, missing information, and deviation of data.

### 2.11 Statistical methods

The primary statistical analyses will be performed by WDB Clinical Research Co., Ltd. using the full analysis data set, from which patients who withdraw consent before assessment of the primary endpoint will be excluded. In addition, we will repeat the analyses in the per-protocol set, which will exclude patients with major protocol deviation. The safety analysis will be performed using the complete data set in the BRIHK group.

For baseline characteristics variables, summary statistics will be calculated using frequencies and proportions for categorical data; means, standard deviations, medians, and quartiles will be used for continuous values. Patient characteristics will be compared using the Chi-squared test or Fisher exact test for categorical valuables and the Wilcoxon rank-sum test for continuous valuables. For the primary intra-group analysis, we will calculate mean HADS and ES-4 scores for each timepoint with 95% confidential intervals and compare the HADS and ES-4 scores at the baseline with those during the treatment period using Wilcoxon signed-rank test. For the inter-group analysis, we will calculate the change in the HADS and ES-4 scores from the pre- to post-treatment period and compare them between the 2 groups using Wilcoxon signed rank test. Then, we will perform an analysis of covariance with the covariates of patient characteristics and calculate the confidence interval. For the analyses of secondary outcome measures between the 2 groups, we will perform the Chi-squared test or Fisher exact test for categorical valuables and Wilcoxon rank-sum test for continuous valuables. *P* values less than 5% will be considered statistically significant, and we will not adjust *P* values in multiple comparisons.

### 2.12 Data monitoring

To oversee the progress of the trial and to ensure that the study is conducted and data are handled in accordance with the protocol and applicable ethical and regulatory requirements, independent data monitor members from the sponsor and competing interests will be appointed. On/off-site risk-based monitoring will be conducted when the first participant is registered, when the 10^th^ participant is registered, and when all of the participants complete the study protocol. On-site monitoring for the storage of data documents will be conducted at both the start and end of this study.

### 2.13 Harms

The adverse events, which include solicited adverse events, spontaneously reported adverse events, and other unintended effects of trial interventions, will be investigated. A follow-up investigation of the adverse events will be conducted, and we will report the outcome to the data and safety monitoring board to assess the causal association with the intervention. Severe adverse events with causal association must be reported to the relevant departments.

### 2.14 Auditing

To assess and assure the reliability and integrity of the study, an independent auditor from the sponsor and competing interests will be appointed. Auditing will be conducted after data collection is completed.

### 2.15 Ethics and dissemination

This trial complies with the Declaration of Helsinki and JP Clinical Trial Act.^[27]^ It was designed as a specified clinical trial and will be conducted by Tohoku University Hospital with approval from the Japanese Ministry of Health, Labour and Welfare certified clinical research review board, Tohoku University (CRB2180001).

### 2.16 Trial status

The protocol and progress are registered on the Japan Registry of Clinical Trials (jRCT s021190001) and University Hospital Medical Information Network (UMIN000031330). Recruitment will begin in April 2019 and is expected to be completed in May 2023. The most recent version of the protocol (ver. 2.1) was approved by the Japanese Ministry of Health, Labour and Welfare certified clinical research review board, Tohoku University (CRB2180001).

## 3 Discussion

In this single-center, randomized controlled clinical trial, we aim to evaluate whether BRIHK is effective for alleviating anxiety and preventing aspiration in the postoperative period in esophageal cancer patients. To date, no clinical trial has been conducted on orally administered drugs, such as Kampo medicine, which could improve anxiety and water brash in the perioperative period of esophageal cancer surgery. The results of this study will be published as a research article, and provide evidence of BRIHK for beneficial mental and physical effects in perioperative esophageal cancer patients.

Hangekobokuto has multiple functions for aspiration, abdominal symptoms, and anxiety, as follows: hangekobokuto administration reduced substance P activity and improved the cough reflex in patients with a history of aspiration pneumonia.^[16]^ Oikawa et al reported that hangekobokuto administration in functional dyspepsia patients decreased abdominal gas volume and symptoms of abdominal pain, indigestion, and constipation.^[28]^ Magnolia officinalis bark, a crude drug component of BRIHK and hangekobokuto, contains an anxiolytic-like agent (4-metylhobokiol) that enhances GABAergic transmission.^[29]^ In addition, we have recently shown that BRIHK ameliorated the delayed gastric emptying induced by corticotropin-releasing factor in a rodent model. BRIHK may not only enhance gastric emptying, but also suppress the corticotropin-releasing factor pathway through acetylcholinesterase, dopamine D2/D3 receptor, and neuropeptide Y Y2 receptor.^[30]^ Therefore, BRIHK could improve aspiration, gastric movement, and anxiety through multiple mechanisms.

In traditional Kampo theory, BRIHK, as well as hangekobokuto, is used for the treatment of “qi stagnation pattern,” which is characterized by a sensation of obstruction in the throat, a sensation of ear-tube obstruction, fullness in the chest, hypochondrium or abdominal distension, a depressive state, or pain.^[31]^ Gastric dysmotility and mental distress in the perioperative period are usually thought to be symptoms of “qi stagnation pattern.” Our study will provide an integrative approach using the standard surgical procedure together with Kampo medicine.

## Acknowledgments

We would like to thank Ms Akiko Kuwabara and Ms Mizue Kusaba for their assistance as clinical research coordinators, Ms Risako Suzuki for the preparation of the test drugs as a hospital pharmacist, Ms Mikiko Fuda for the measurement of physical nutritional status as a dietitian, and Dr Shin-ichi Fujimaki for the preparation of blood samples. We would like to thank Editage (www.editage.com) for English language editing.

## Author contributions

**Conceptualization:** Shin Takayama and Tadashi Ishii.

**Funding acquisition:** Shin Takayama.

**Investigation:** Ryutaro Arita, Shin Takayama, Hiroshi Okamoto, Ken Koseki, Yusuke Taniyama, Takashi Kamei.

**Methodology:** Ryutaro Arita, Shin Takayama, Soichiro Kaneko, Takashi Kamei.

**Project administration:** Ryutaro Arita, Shin Takayama, Tadashi Ishii.

**Resources:** Shin Takayama, Takashi Kamei, Tadashi Ishii.

**Supervision:** Shin Takayama, Hiroshi Okamoto, Soichiro Kaneko, Takashi Kamei and Tadashi Ishii.

**Writing – original draft:** Ryutaro Arita.

**Writing – review & editing:** Ryutaro Arita, Shin Takayama, Hiroshi Okamoto, Ken Koseki, Yusuke Taniyama, Rie Ono, Satoko Suzuki, Tadashi Ishii.
